# Genomic profiling and experimental validation of type VI secretion system-associated proteins in *Klebsiella*

**DOI:** 10.1371/journal.pgen.1011878

**Published:** 2025-09-19

**Authors:** Ning Zhu, Yuzhe Zhao, Wenjing Yi, Lili Jiang, Tingting Dong, Zhihan Peng, Shanshan Jia, Ruihong Wu, Xiaohan Guo, Arnab Pain, Lei Song, Qingtian Guan

**Affiliations:** 1 Bioinformatics Laboratory, Center for Infectious Diseases and Pathogen Biology, The First Hospital of Jilin University, Changchun, China; 2 Department of Respiratory Medicine, Center for Pathogen Biology and Infectious Diseases, Jilin Provincial Key Laboratory for Individualized Diagnosis and Treatment of Pulmonary Diseases, The First Hospital of Jilin University, Changchun, China; 3 Health Examination Center, The First Hospital of Jilin University, Changchun, China; 4 The Core Facility of the First Hospital of Jilin University, Changchun, China; 5 Pathogen Genomics Laboratory, Biological and Environmental Sciences and Engineering, King Abdullah University of Science and Technology, Jeddah, Saudi Arabia; Uppsala University: Uppsala Universitet, SWEDEN

## Abstract

*Klebsiella* is widely distributed in nature and recognized for its dual role as a human commensal and an opportunistic pathogen capable of causing severe infections. Among its key virulence mechanisms, the Type VI Secretion System (T6SS) plays a critical role in microbial competition, interspecies interactions, and environmental adaptation. In this study, we investigated a representative set of high-quality *Klebsiella* genomes and identified two major T6SS loci (T6SS^*kleb*1^ and T6SS^*kleb*2^), which exhibit distinct structural features and are predominantly found within the *Klebsiella pneumoniae* species complex (KpSC). Comparative genomics further revealed the diverse distribution of effector and immunity proteins and their correlation with T6SS loci. Based on this distributional bias, we developed a novel computational approach to identify protein families significantly associated with T6SS. These T6SS-associated gene clusters were implicated in mediating adhesion to host cell surfaces during urinary tract infections, as well as in metabolism and immune defense. Moreover, we identified three putative orphan effectors harboring DUF3258, DUF3751, and Sel1 domains. Overexpression of these proteins in *Escherichia coli* resulted in cytotoxic effects, supporting their potential as T6SS effectors. These findings establish a comprehensive framework for T6SS analysis, aiming to expand and enrich our understanding of the diversity, evolutionary trajectory, and functional implications of T6SS in *Klebsiella*.

## Introduction

The genus *Klebsiella*, a group of Gram-negative, oxidase-negative, rod-shaped bacteria, that are normally found as normal flora in the nose, mouth, and gastrointestinal tract; however, they can also act as opportunistic human pathogens. *Klebsiella* organisms can cause various disease states, notably pneumonia, urinary tract infections, sepsis, meningitis, diarrhea, peritonitis and soft tissue infections [1]. Among species of the genus *Klebsiella*, *K. pneumoniae* is the most prevalent. A significant proportion of infections caused by *K. pneumoniae* can be attributed to two primary pathotypes: multidrug-resistant (MDR) and hypervirulent (hv) clones [2]. Also, *K. pneumoniae* has demonstrated the ability to acquire mobile elements and genetic rearrangements that transfer antimicrobial resistance and/or virulence traits, leading to simultaneously exhibit hypervirulence and anti-microbial resistance, termed MDR-hv *K. pneumoniae* [3]. The increasing growth of serious infections due to hypervirulence and ineffective treatment caused by antibiotic resistance rendered MDR-hv *K. pneumoniae* a real superbug that poses a serious threat to public health [4]. Apart from *K. pneumoniae*, *Klebsiella oxytoca* is also a major source of community-acquired pneumonia and nosocomial infections and the second most common cause of gram-negative bacteremia and urinary tract infections in Asia [5].

One of the critical factors contributing to the pathogenicity of *Klebsiella* species is the Type VI Secretion System (T6SS), a complex nanomachine used by many Gram-negative bacteria to deliver effector proteins directly into target cells. Initially identified in *Vibrio cholerae* [6], the T6SS is composed of several components that form a phage-like structure anchored to the bacterial membrane and usually consists of 13 core components designated TssA-M. The core structure of T6SS resembles an inverted bacteriophage tail, containing membrane complex, baseplate, and tail tube/sheath complex [7]. The membrane complex is composed of three proteins, TssJ, TssL, and TssM are together involved in anchoring the system to the bacterial inner membrane and facilitating the assembly of the rest of the structure. Similar to the baseplates of contractile phages, the T6SS baseplate comprises a central hub surrounded by at least six proteins, which is essential for the assembly of the TssD tube and sheath and triggering sheath contraction. TssB and TssC constitute the contractile sheath of T6SS, which encases the inner TssD tube during assembly [7,8]. The T6SS is predominantly associated with antagonistic activities, mediating both inter- and intra-bacterial competition [9–11]. Its effector proteins exert antimicrobial activity by targeting peptidoglycan, bacterial nucleic acids, and disruption of cell membranes [12]. Recent studies have broadened the original understanding of T6SS effector proteins, demonstrating their roles not only in bacterial competition, but also as potent weapons against fungi and eukaryotic cells. For example, the first fungal-specific T6SS effector proteins, Tfe1 and Tfe2 from *Serratia marcescens*, showing antifungal activity against *Saccharomyces cerevisiae* and *Candida glabrata* [13,14]. Immunity proteins are typically encoded adjunct to effector proteins mediating antimicrobial activity to antagonize self-intoxication [15,16].

Effector protein identification in T6SS has been achieved through both experimental and computational approaches. Experimental approaches, such as comparative proteomics [11,17,18], transposon mutagenesis [19] have successfully identified effectors with antimicrobial and anti-eukaryotic activities in several bacterial species. However, these methods are labor-intensive, often require specific conditions to induce T6SS activity, and may fail to detect lowly expressed effectors under particular environmental or physiological contexts. Computerized approaches, such as analysis of genes within the T6SS cluster and auxiliary modules [20–22], as well as genes encoding T6SS marker domains (e.g., MIX and FIX) [22,23], demonstrate the potential to use such tools for discovering new toxins. Nevertheless, current approaches-whether experimental or computational-tend to focus on effectors encoded within T6SS loci or immediately downstream of *vgrG*, potentially overlooking orphan genes located outside these regions. In this context, Fridman *et al*. introduced a methodology relying on a comparative genomics that enabled the discovery of a widely distributed, membrane-disrupting effector family in *Vibrio parahaemolyticus* [24]. Building on this strategy, and to complement both traditional experimental methods and existing computational pipelines, we have developed an integrative framework combining comparative genomics, bioinformatic screening, and experimental validation. This approach enables the systematic identification of orphan T6SS effectors in *Klebsiella* species and provides a novel model for uncovering previously unrecognized components of the T6SS arsenal.

Current studies on the genus *Klebsiella* have focused predominantly on *K. pneumoniae*, while often overlooking other species within the genus. Moreover, research on *K. pneumoniae* has frequently been limited to a small number of clinical strains or has lacked comprehensive genomic analyses. Although T6SS was known to contribute to bacterial competition, cell invasion and colonization in *K. pneumoniae* [9], significant gaps remain in our understanding of the distribution and diversity of this system in *Klebsiella*, necessitating further investigation. This study addresses these gaps by comprehensively analyzing the genomes of all species within the genus *Klebsiella*, characterizing the genomic distribution and diversity of T6SS loci, employing statistical and computational approaches to predict effector proteins and identify putative candidates, and performing experimental validation to confirm the antagonistic activity and functional roles of key effectors. This work enhances our understanding of the genomic diversity and distribution of T6SS in *Klebsiella*, shedding light on its role in bacterial competition and species-specific adaptations.

## Results

### Identification and characterization of T6SS loci and phylogeny analysis in *Klebsiella* spp.

**Fig 1:**
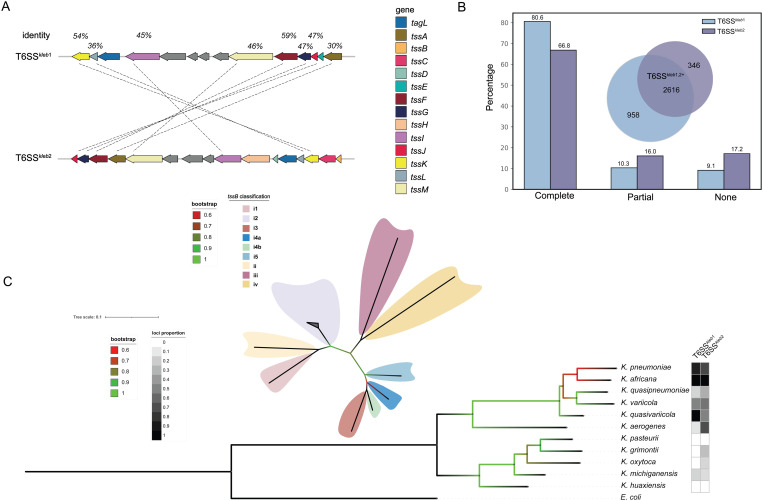
Distribution and characterization of T6SS loci across the *Klebsiella* genus. (A) Genomic organization of two representative *Klebsiella* genomes displaying T6SS gene clusters. Core T6SS structural proteins are shown in colored arrows, and non-core proteins in gray. Percent identity between homologous proteins was calculated using BLASTp (default NCBI parameters) and is indicated above connecting dashed lines. Gray arrows indicate non-core regional proteins. Percent identity between homologous proteins was calculated using BLASTp with default NCBI parameters. (B) Percentage of genomes harboring complete, partial, or no T6SS^*kleb*1^ or T6SS^*kleb*2^ gene clusters. The Venn diagram shows the number of genomes containing one or both complete gene clusters. (C) Maximum-likelihood phylogenetic tree built from 11 *Klebsiella* species using 10,000 bootstrap replicates, with *E. coli* as the outgroup. The two columns on the right indicate the proportion of genomes with complete T6SS^*kleb*1^ or T6SS^*kleb*2^ gene clusters for each species. An unrooted phylogenetic tree of the core protein TssB was also constructed, showing nine distinct groups, with 139 representative TssB sequences from *Klebsiella* genus. All TssB sequences within *Klebsiella* belong to the i2 type, and branches have been collapsed for clarity.

The high-quality genome that passed the quality control (S1 Table) were compared against the protein database obtained from SecReT6 [25] using Proteinortho v 6.3.0 [26] to identify T6SS loci based on the T6SS core components and other regionally intrinsic proteins. The distribution of T6SS of total 4,434 genomes from the NCBI databases was evaluated and two major types of T6SS loci in *Klebsiella* genus have been identified, here, namely, T6SS^*kleb*1^ and T6SS^*kleb*2^, respectively. To assess the similarity between homologous genes, a BLASTn alignment was performed, and the results suggested that these two T6SS loci are not derived from a single ancestral locus through duplication event (Fig 1A).

Notably, loci T6SS^*kleb*2^ does not contain any *tssE*, which participates in the composition of baseplate, and T6SS^*kleb*1^ lacks several core components, including *tssB*, *tssC*, *tssD* and *tssH*. We observed that genes encoding *tssD* (11,544/14,160) and *vgrG* (4,366/11,195) were situated outside the T6SS locus among the T6SS^*kleb*1,2^ +genomes, and putative effectors are commonly encoded in the vicinity of these “orphan” genes which have also been observed in other studies [27,28]. Furthermore, the *vgrG* genes in both of the loci are located at a relative variable region where various effectors and immunity proteins were located, as also reported by previous studies [27,29].

Based on the classification of gene clusters as complete or incomplete, the majority of these strains contained complete T6SS loci encoding conserved genes, which accounted for 3,574/4,434 for T6SS^*kleb*1^ and 2,962/4,434 for T6SS^*kleb*2^, respectively. Additionally, 2,616 strains contained two complete gene loci within their genomes. In contrast, a total of 860 and 1,472 genomes which have incomplete T6SS^*kleb*1^ and T6SS^*kleb*2^ (Fig 1B).

In addition to the core components of the T6SS gene clusters, TagL has been identified in the formation of both of the loci. TagL acts as a component of the basal membrane complex to help anchor to the cell wall. It is worth noting that TagL Peptidoglycan-binding domain (PGBD) stabilizes the transmembrane complex of the T6SS by binding to peptidoglycan, thereby helping to withstand the forces generated during sheath contraction [30].

A phylogenetic tree was constructed to investigate the distribution of T6SS loci in relation to species phylogeny. The strains were mainly grouped into two phylogroups, and with T6SS^*kleb*1^ is notably more abundant in *K. pneumoniae* monophyletic group, which corresponds to species within the KpSC, including *K. pneumoniae*, *Klebsiella quasipneumoniae*, *Klebsiella variicola*, *K. quasipneumoniae* subsp. *similipneumoniae*, *K. variicola* subsp. *tropica*, *Klebsiella quasivariicola*, and *Klebsiella africana*. In contrast, species within the *Klebsiella huaxiensis* clade exhibited a lower number of T6SS loci. The prevalence of T6SS^*kleb*1^ and T6SS^*kleb*2^ in these species was markedly reduced (Fig 1C), with respective frequencies ranging from 0% to 17% and 0% to 25% of each species.

Moreover, based on the schemes proposed by Barret *et al*. [31,32] and Russell *et al*. [33] from SecReT6 [25], T6SS could be classified into four types according to the *tssB* genes, namely, T6SS^i^, T6SS^ii^, T6SS^iii^ and T6SS^iv^, with six subtypes (i1, i2, i3, i4a, i4b and i5) have been identified within T6SS^i^ system. In our study, a total of 2,962 complete genomes containing *tssB* were obtained, of which 139 representative sequences after clustering were used for further classification. The type i2 has been identified in all strains with complete T6SS^*kleb*2^ cluster in *Klebsiella* spp. except for *Klebsiella pasteurii* and *K. huaxiensis* (Fig 1C), which do not possess any T6SS^*kleb*2^, enriching the knowledge of TssB classification for *Klebsiella*.

### Comprehensive analysis of T6SS effectors and immunity proteins across *Klebsiella* genus reveals correlation with complete T6SS loci

**Fig 2:**
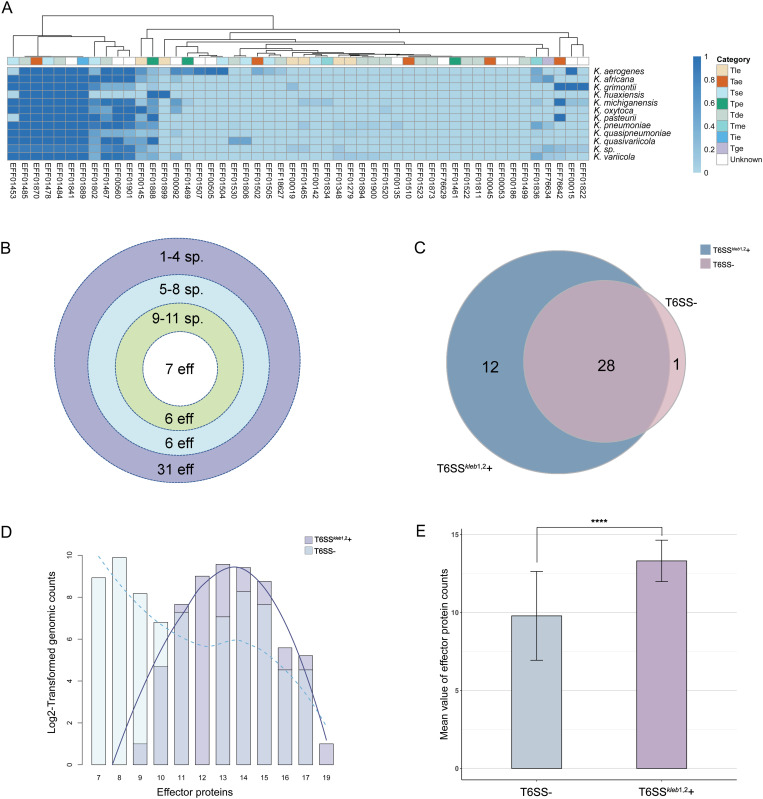
Distribution of T6SS effector proteins across *Klebsiella* species. (A) Heatmap showing the proportions of 50 effector proteins in the genomes of various *Klebsiella* species. (B) Concentric rings represent the distribution of effector proteins (eff) across different species. The innermost ring represents 7 effector proteins found across all species. (C) Comparison of unique effector proteins between genomes with T6SS^*leb*1,2^+ and genomes without any T6SS loci (T6SS-), illustrated by a Venn diagram. (D) Fold-change analysis (log2-transformed) of genomic counts for effector proteins between T6SS^*kleb*1,2^+ and T6SS- genomes, with a fitted trend line. Effector proteins are labeled numerically. (E) Comparison of the mean value of effector proteins per each genome between T6SS^*kleb*1,2^+ (n = 2,616) and T6SS- (n = 190). Error bars represent the standard deviation, and statistical significance was assessed using a one-sided Welch’s t-test (****p < 0.0001).

To gain deeper insights into the T6SS effectors and immunity proteins, we performed a comprehensive homologous search across 4,434 genomes with experimentally validated T6SS effectors (T6SEs) and immunity proteins (T6SIs). A total of 50 effector protein groups and 28 immunity protein groups have been identified across genus (Figs 2A and S1A) with notable heterogeneous in their distribution. Additionally, we identified a total of 8 distinct categories along with corresponding structural domains of effector proteins including peptidase effector (Tpe), DNase and RNase effector (Tde), lipase effector (Tle), amidase effector (Tae), membrane-disrupting effector (Tme), metal iron acquisition effector (Tie), glycoside hydrolase effector (Tge) and unclassified secretion system effector (Tse) (Table 1). Seven effector and four immunity proteins were found across all *Klebsiella* species (Figs 2B and S1B). Notably, 10 effectors (EFF01499, EFF00186, EFF00053, EFF00045, EFF01811, EFF01522, EFF01461, EFF76629, EFF01873, EFF01523) were exclusively found in *K. pneumoniae.* The immunity proteins displayed a more homogeneous and less species-specific distribution compared to the effectors.

**Table 1:** Identified effectors in *Klebsiella* spp.

Category	Effectors	Identified domains
Tse	EFF01806, EFF00142, EFF01453, EFF01478, EFF01504, EFF01505, EFF01802	Mn_catalase; CSD; RNAse_A_bac; rve, rve_2, HTH_Tnp_1, HTH_21;
Tpe	EFF01888, EFF01461, EFF01469	Not detected
Tde	EFF01467, EFF01484, EFF01485, EFF01499, EFF01520, EFF01522, EFF01523, EFF01530, EFF01811, EFF01873, EFF01894, EFF01900, EFF18627	Phage_GPD, Pyocin_S, DUF2345, T6SS_Vgr, Phage_base_V; BON; tRNA-synt_1e, DALR_2; PAAR_motif, DUF6531, RHS, RHS_repeat, RHS_repeat, RHS_repeat, RHS_repeat; T6SS_HCP; Pyocin_S, T6SS_HCP; Usp; Pyocin_S, PAAR_motif; Imm52; Tox-REase-5; AHH, RHS;
Tle	EFF00145, EFF01279, EFF01348, EFF01465, EFF00019, EFF01348, EFF01899	DUF2235, DUF2235; PLDc_2; PGAP1; PLDc, PLDc;
Tae	EFF00045, EFF01502, EFF01510, EFF01870, EFF76642	Tae4; Amidase_2; Amidase, Amidase;
Tme	EFF01834, EFF01836	Not detected
Tie	EFF01889	SBP_bac_11
Tge	EFF76634	Peptidase_M23, LysM;

Tse, unclassified secretion system effector; Tpe, T6SS peptidase effector; Tde, T6SS DNase/RNase effector; Tle, T6SS lipase effector; Tae, T6SS amidase effector; Tme, T6SS muramidase effector; Tie, T6SS metal iron acquisition effector; Tge, T6SS glycoside hydrolase effector.

We hypothesis that the genomes with a complete T6SS locus exhibit a higher abundance of T6SS-associated genes than the genomes without any. To test the hypothesis, we conducted a comparative genomics analysis between the strains with genomes with either both or without any T6SS loci (T6SS^*kleb*1,2^ + /2,616genomes, T6SS-/190genomes), with detailed definitions provided in the Methods section. For effectors, 40 types were identified in the T6SS^*kleb*1,2^ + genomes, whereas only 29 were found in the T6SS- genomes (Fig 2C). The distribution of T6SS effectors showed a skew distribution with a higher number of effectors in the T6SS^*kleb*1,2^ + group compared with the T6SS- group (Fig 2D). Similarly, the Welch’s t-test revealed that the mean value of effectors in T6SS^*kleb*1,2^ + group is significantly higher than that in T6SS- group (*p* < 0.0001) (Fig 2E).

Meanwhile, comparisons of immunity proteins between the two groups yielded results consistent with effectors that the immunity proteins are more prevalent in the T6SS^*kleb*1,2^ + group (S2 Fig). These results indicated that T6SS effectors and immunity proteins are widely distributed across the genus with various distribution, and the statistical significance supports the hypothesis that the presence of a complete T6SS locus correlates with a greater abundance of T6SS-related genes.

### Categorization of T6SS effectors and immunity proteins downstream of *vgrG* in *Klebsiella* species

**Fig 3:**
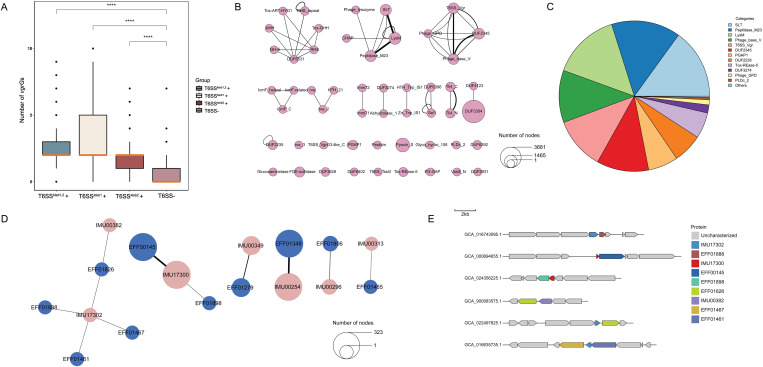
Characterization of structural domains in genes downstream of *vgrG*s. (A) Boxplot comparing the number of *vgrG* genes across four genome groups: T6SS^*kleb*1,2^ + , T6SS^*kleb*1^ + , T6SS^*kleb*2^ + , and T6SS- genomes. ****p < 0.0001 (Mann-Whitney U Test). Black circles represent outliers. Median lines are shown in a uniform color to improve visibility when overlapping with box borders. (B) Structural annotation of proteins downstream of *vgrG*s. The network diagram shows connections between domains in downstream proteins. Circle size represents the relative abundance of each domain, and line thickness indicates the frequency of specific domain combinations. (C) Pie chart summarizing the classification of structural domains of effector proteins based on experimentally validated data from the SecReT6 database. Domains with less than 1% relative abundance were grouped under “Others”. (D) Network analysis of three downstream genes annotated as identified effector and immunity proteins. Genes that are adjacent on the same genome are linked. The size of the circle represents the abundance of certain domain, and thickness of the connected line indicates frequency of particular connection. (E) Schematic representation of gene clusters for genes encoding effectors and immunity proteins. Gray arrows indicate genes encoding other regional proteins other than T6SS effectors or immunity proteins.

In fact, the C-terminal tail of the VgrG protein plays a critical role in determining the specificity of effector recognition and delivery [34]. Notably, orphan *vgrG* and *tssD* genes—those located outside the main T6SS cluster—are often found in the proximity of genes encoding putative effector proteins [28]. Previous studies have also shown that effectors and cognate immunity proteins are often encoded downstream of *vgrG*s [35]. For these reasons, *vgrG* often serves as an essential resource for effector identification. Therefore, to systematically identify effectors associated with *vgrG*s, explore their relationships with immunity proteins, and characterize domains associated with these genes, we analyzed up to three downstream genes in all T6SS^*kleb*1,2^+ and T6SS- genomes. As a first step, we compared the number of *vgrG*s between T6SS^*kleb*1,2^+ and T6SS- genomes. As expected, the number of *vgrG* genes is significantly lower in genomes without any loci compared with those containing one or both of the loci (*p* < 0.001) (Fig 3A). Each T6SS^*kleb*1,2^ + genome contains 2.51 *vgrG* genes while the T6SS- genomes possess 0.70 *vgrG*s on average. Moreover, 13,736 proteins downstream of *vgrG*s were identified, among which 5,305 have been experimentally validated as homologs in the SecReT6 [25] database.

Analysis of the genes downstream of *vgrG*s revealed several highly frequent functional domains associated with both effector and immunity proteins (Fig 3B), along with functional domains characteristic of experimentally validated effector proteins (Fig 3C). Among these, DUF3304 is the most abundant, appearing 3,681 times across all analyzed proteins. Other frequently observed domains included Pyocin_S (1,465 occurrences), LysM (991 occurrences), and Peptidase_M23 (809 occurrences). DUF3304 domain was found in several known bacterial immunity proteins [36–38] (S2 Table), suggesting their potential role in effector-immunity interactions while the exact function of the domain remains to be uncharacterized. The consistent presence of Pyocin_S, LysM, and Peptidase_M23 highlights their widespread use in bacterial competition strategies. Additionally, SLT transglycosylase (801 occurrences), primarily involved in cell wall degradation, was also commonly identified.

Network analysis of the structural domains identified recurring combinations in effector proteins, reflecting their modular architecture. Here, we operationally define a “modular architecture” as a recurrent pattern of connected protein domains, based on their co-occurrence in individual proteins. For example, effector proteins frequently included RHS domains in combination with DUF6531, GH-E, AHH, Tox-GHH, Tox-ART-HYD-1 forming large multi-domain toxins with diverse functionalities. Similarly, LysM, Peptidase_M23, and SLT were often co-localized within degradative effectors, underscoring their role in targeting bacterial cell walls within the genus. Phage-derived domains, such as Phage_base_V and Phage_GPD, were also prevalent among the identified effectors. These domains reflect the evolutionary relationship between T6SS and bacteriophage tail-like structures, as previous studies have proposed that T6SS machinery shares a common evolutionary origin with phage contractile systems [8,39].

The relationship between effectors and immunity proteins demonstrated notable complexity and flexibility. A single effector can associate with multiple immunity proteins, and conversely, a single immunity protein can cognate with different effectors (Fig 3D and 3E). For instance, IMU17302, whose cognate effector was identified as EFF01888 (Tpe) in *Aeromonas dhakensis* strain SSU [40], was also found adjacent to multiple effectors, including EFF01826 (Tpe), EFF01467 (Tde), and EFF01461 (Tpe). Meanwhile, EFF01826 was shown to cognate with IMU00349, highlighting the dynamic interplay between effectors and immunity proteins.

### Identification of T6SS-associated orthologous groups

To establish a baseline for the association between T6SS loci and T6SS-associated genes, we first compared genomes harboring both T6SS^*kleb*1^ and T6SS^*kleb*2^ loci (T6SS^*kleb*1,2^+) with genomes lacking both loci (T6SS-). This comparison revealed a clear enrichment of known T6SS-associated proteins in T6SS+ genomes (Fig 2E). Building on this, we conducted two independent locus-specific analyses: T6SS^*kleb*1^ + vs T6SS^*kleb*1^- (regardless of T6SS^*kleb*2^ status), and T6SS^*kleb*2^ + vs T6SS^*kleb*2^- (regardless of T6SS^*kleb*1^ status). These comparisons aimed to identify orthologous groups that are specifically associated with each individual locus. For completeness, we also performed alternative comparisons between T6SS ^*kleb*1,2^ + vs T6SS^*kleb*1+,2−^ or T6SS^*kleb*2+,1−^, which may reflect cumulative or combinatorial effects. These additional comparisons are provided in S3 Fig and S3 Table. A total of 89,182 and 84,859 protein families were clustered for the genomes containing T6SS^*kleb*1^ + /T6SS^*kleb*1^- and T6SS^*kleb*2^ + /T6SS^*kleb*2^- and the orthologous groups that are significantly associated with T6SS were identified (see Methods for the details). Overall, 608 orthologous groups are significantly associated with T6SS^*kleb*1^ and 377 are significantly associated with T6SS^*kleb*2^. The protein families were further annotated with SecReT6 [25,41] database, Bastion6 [42] and eggNOG-mapper v2 [43] as listed in S2 Table. A network analysis was performed to study the interaction of those protein groups which revealed that the orthologous groups are well connected in the genomes.

Based on the results from various annotations, known T6SS-associated proteins, core-components, potential effectors, and unknown functional gene clusters were identified among the T6SS-associated protein families. Known proteins associated with the T6SS include components of the incomplete locus itself, as well as related effector and immunity proteins. Furthermore, several potential novel T6SS effectors have also surfaced.

**Fig 4:**
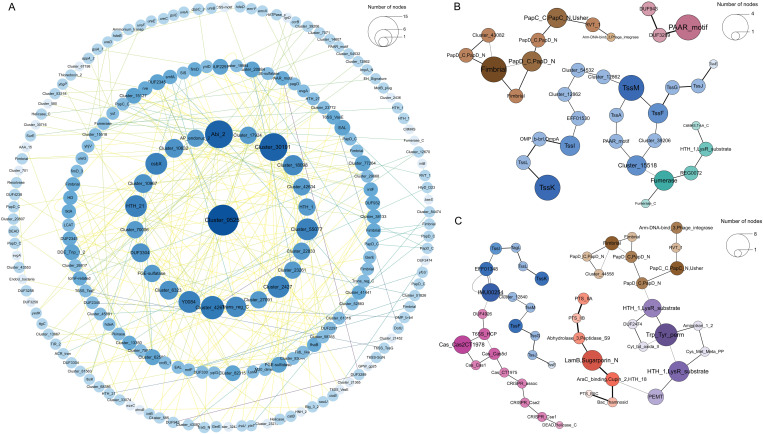
Network analysis of protein families enriched in T6SS^*kleb*1/2^-associated genomes. (A) The network, constructed using Cytoscape, includes 377 protein families associated with T6SS^*kleb*2^. Node size is proportional to the number of proteins in each family, with colors transitioning from dark to light based on abundance. Edge colors transition from yellow to green to purple based on interaction frequency. Node labels are prioritized by preferred names, followed by eggNOG-mapper annotations and cluster IDs. Representative loci of (B) T6SS^*kleb*2^ and (C) T6SS^*kleb*1^-associated protein families. Gene clusters are depicted with consistent color hues for proteins located within the same locus or sharing related biological functions. Node size represents the number of proteins within a family, and colors transition from dark to light based on abundance. Line thickness reflects the frequency of protein-protein interactions.

Cluster9525 and Cluster986 (DUF2345 domain containing protein), which contain the highest number of proteins as shown as central node in the interaction network in T6SS^*kleb*2^ and T6SS^*kleb*1^ respectively (Figs 4A and S4), likely play important roles in the biological functions associated with T6SS. Notably, DUF2345 domain can be encoded either as an independent gene or as part of other proteins, being fused to the C-terminus of VgrG or the N-terminus of toxin, and it has been experimentally demonstrated that proteins containing DUF2345 assist the interaction between VgrG and corresponding effectors, which is critical in the secretion process of T6SS effectors [35]. However, the protein family in which Cluster9525 lacks definitive structural annotation, necessitating further research to confirm its potential role in T6SS.

Some putative EI pairs also caught our attention. In T6SS^*kleb*2^, Cluster_51573 and Cluster_10480 show high linking frequencies (n = 780), with Cluster_10480 containing DUF3289 and Cluster_51573 containing DUF943 which was predicted as T6SE and non-T6SE by Bastion6 [42] respectively among the identified protein families (Fig 4B and S2 Table). Coincidentally, a recent study that used Alphafold-Multimer-based regression model have predicted the same DUF3289-containing protein as a T6SS effector. This study demonstrated that bacterial cells expressing the effector candidate T6EC1 (DUF3289 domain containing protein) in their peripheral plasma changed to a rounded morphology, indicative of cytotoxicity. In contrast, cells co-expressing T6EC1 and its cognate immunity protein T6IC1 (containing a DUF943 domain) maintained their characteristic rod shape [44]. This overlapping discovery further strengthens the reliability of our predictions.

In addition to the putative orphan effector proteins, several gene clusters consisting of genes located adjacent to one another within the same genome have also been identified (Figs 4B, 4C, and S5 and S4 Table). Not surprisingly, core-components of the T6SS locus were unequivocally identified in both T6SS^*kleb*2^ and T6SS^*kleb*1^(Fig 4B and 4C). Furthermore, we discovered a widely enriched gene cluster that facilitates bacterial adhesion to host cell surfaces in both T6SS^*kleb*1^ and T6SS^*kleb*2^ among the protein families enriched in T6SS+ genomes. This system, consisting of PapC_C, PapC_N, and Usher-containing proteins, constitutes the bacterial “Usher-Chaperone” system, which plays a critical role in mediating adhesion to host cell surfaces, particularly during the early stages of urinary tract infections [45]. Synergistically, Fimbrial containing proteins contribute to bacterial virulence by mediating surface adhesion, promoting biofilm formation, and enabling self-aggregation-critical processes that are critical for establishing interactions with host environments [46].

Additionally, among the protein families that are associated with T6SS^*kleb*1^ are several gene clusters associated with basal metabolism and immune defense (Fig 4C). For example, proteins with PTS_EIIC, PTS_IIB, and PTS_IIA participate in sugar metabolism and transport [47], while those containing Aminotran_1_2 and Cys_Met_Meta_PP domains are involved in the metabolism of amino acids and sulfur-containing compounds [48], and proteins containing HTH_1 and LysR_substrate domains regulate the gene transcription. Lastly, proteins containing CRISPR_Cse1, CRISPR_Cse2, and related domains form a gene locus significantly associated with T6SS, suggesting a coordinated role in bacterial defense and environmental adaptation [49,50]. In fact, recent studies have revealed potential interactions between the T6SS and CRISPR-Cas systems at both regulatory and genomic levels. In *Aliivibrio wodanis*, quorum sensing (QS) regulators, particularly LitR, coordinately control the expression of both T6SS and a type I-F CRISPR-Cas system, with T6SS1/2 and CRISPR genes being upregulated at high cell density and low temperature [51]. Meanwhile, in *Vibrio cholerae*, a complete CRISPR-Cas system and a T6SS gene cluster are co-localized on the excisable pathogenicity island VPI-6, which contains conserved recombination modules, suggesting a potential evolutionary linkage and cooperative defense strategy [52]. These findings support the view that T6SS and CRISPR-Cas may function in coordination to respond to environmental challenges and genetic threats. We have also observed a statistically significant difference in Fumarase-containing proteins in T6SS^*kleb*2^ + vs T6SS^*kleb*2^-, although its biological correlation to T6SS integrity remains unclear.

### Proteins harboring DUF3258, DUF3751, and Sel1 domains were toxic to *E. coli*

Among the T6SS-associated protein families, we applied a tiered selection strategy based on bioinformatic filtering, experimental feasibility, and scientific relevance to refine our candidate pool. We excluded families previously validated as T6SS-related, those with known annotated functions, and those predicted as “no” by Bastion6 (S2 Table). To ensure experimental tractability, we prioritized candidates encoded in our clinical *K. pneumoniae* isolates, which facilitated downstream assays. This filtering yielded a smaller set of high-confidence candidates with distinct features warranting further investigation. The orthologous group Cluster_28032 includes DUF3258 (pfam11646) and the phage_integrase domain, which is essential for the integration and excision of bacteriophage DNA into and from a host genome during bacterial infection. Cluster_4724 contains DUF3751 (pfam12571) and the phage_fiber_2 domain, which is responsible for phage attachment to host cells. Sel1, found in Cluster_8841, is associated with two Sel1-like proteins, RS09150 and RS09155 in *Salmonella pullorum*, which have been shown to promote the expression of T3SS genes and contribute to bacterial infection in chickens [53]. Importantly, two candidates contain DUF3258 or DUF3751 domains, which are entirely novel and uncharacterized, while the Sel1-like domain, previously linked to T3SS, is identified here for the first time as a potential T6SS effector. Taken together, these three groups demonstrate significant in silico association with T6SS and potential cytotoxic activity, prompting subsequent experimental investigation to explore their functional roles.

**Fig 5:**
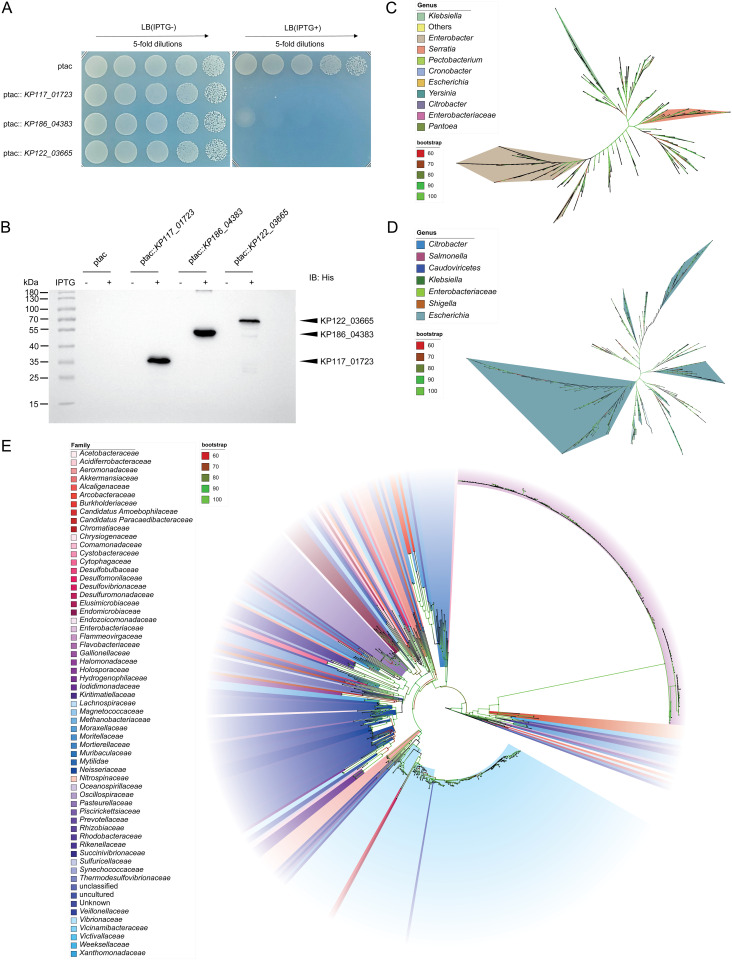
Expression of proteins harboring DUF3258, DUF3751, and Sel1 domain in *E. coli* and their homologs distribution across taxa from different domains of life. (A) Drop assay showing a marked reduction in the growth of the *E. coli* DH5α expressing ptac empty vector (control), KP117_01723, KP186_04383, and KP122_03665. Exponential-phase cultures of *E. coli* expressing different proteins were adjusted for OD600 and serial diluted onto LB media supplemented with and without 0.2mM IPTG. The cells were prepared with serial 5-fold dilutions from left to right. (B) Western blot assay using an anti-His monoclonal antibody (IB: His) demonstrating the expression of KP117_01723, KP186_04383, KP122_03665 in *E. coli* DH5α with 0.2mM IPTG. Control with the ptac promoter only, with and without IPTG induction. (C) Unrooted phylogenetic tree was constructed by IQ-TREE with 10,000 bootstrap replicates of the protein containing DUF3258 domain at the genus level, in which the highlighted colors presented represent the three most distributed genera. Genera that accounted for less than 5 were combined into one group “Others”. (D) Unrooted phylogenetic tree was constructed by IQ-TREE with 10,000 bootstrap replicates of the proteins containing DUF3751 domain at the genus level. *Escherichia* spp. accounts for 90% of all genera, are predominantly represented. (E) Maximum-likelihood tree with 10,000 bootstrap replicates was constructed for protein containing Sel1 domain at the family level, with tip labels indicating strains after three iterations of PSI-blast (with an expect value threshold of 5 × 10^−3^ in each iteration).

Consequently, we cloned the genes *KP117_01723*, *KP186_04383*, *KP122_03665* whose protein containing the DUF3258, DUF3751, and Sel1 domain respectively into an inducible expression vector and transformed this construct into the *E. coli* strain DH5α. Following induction with IPTG, we observed a marked reduction in the growth of the *E. coli* strains expressing these proteins compared to the control carrying the empty vector, with the extent of growth inhibition varying among the different proteins (Fig 5A). Western blot analysis using a mouse anti-His monoclonal antibody confirmed these proteins were successfully expressed in *E. coli* upon IPTG induction. No protein was detected in the empty vector control (ptac), confirming specificity of expression. (Fig 5B). These findings provide evidence that the proteins identified via our bioinformatics approach are likely candidates for T6SS substrates.

To further clarify the distribution of these proteins containing above structural domains in nature, multiple sequence alignment of their homologs, identified in PSI–BLAST. The phylogenetic analysis at the genus level of KP117_01723 and KP186_04383 indicates that they were predominantly distributed in prokaryotes (Fig 5C and 5D). Specifically, KP117_01723 is mainly found in the *Enterobacter* (181/500) and *Klebsiella* (113/500), while KP186_04383 is primarily associated with the genus *Escherichia* (457/500). All genomes encoding KP117_01723 possessed complete T6SS locus, and 50% of these genomes contain at least one *tssD* gene. In contrast, KP186_04383-encoding genomes is exclusively harbored T6SS^*kleb*1^ (*tssD* gene was missing) and 94.3% of the genomes at least harbored one *vgrG* gene, together with *tssD*, which are hallmark T6SS-secreted tail tube components. Analysis of the phylogenetic distribution of KP122_03665 revealed a more diverse family (Fig 5E). The bootstrap values for the major branches containing these three proteins are all above 80, supporting the robustness of the inferred phylogenetic relationships. Genomes containing KP122_03665 at the family level were prevalent in *Vibrionaceae* and *Enterobacteriaceae*. Remarkably, 99% genomes encoding KP122_03665 also harbored T6SS locus (containing at least 8 out of 13 T6SS core components that were shown to specifically predict T6SS), and 52.1% of the genomes harbored at least one *tssD* gene. Moreover, Eukaryotes like *Mytilidae* and *Gallionellaceae* have also been found, which is not surprising that previous study showed the eukaryotic Sel1 proteins are involved in the ER-associated protein degradation [41]. These results suggest KP117_01723, KP186_04383 and KP122_03665 proteins are predominantly associated with T6SSs. Further support for this conclusion was revealed by the experimental results described above.

## Discussion

In this study, we conducted a comprehensive analysis of the T6SS including core structural components, effectors and immunity proteins in *Klebsiella* genus. We investigated the genes families that are significantly enriched in the presence of T6SS, and validate novel putative effectors in *Klebsiella* spp*..* We found that majority of *Klebsiella* strains possess a complete T6SS apparatus, organized into two distinct loci, designated as T6SS^*kleb*1^ and T6SS^*kleb*2^. Similar locus architectures have previously been identified in specific strains, such as *K. pneumoniae* CH1157 [54] and HS11286 [55]. Notably, T6SS^*kleb*1^ differs from T6SS^*kleb*2^ in that it does not encode essential T6SS core components such as TssB, TssC, TssD, and TssH. The low identity between orthologous proteins at these loci suggests that they were not derived by a duplication event.

The two T6SS loci, T6SS^*kleb*1^ and T6SS^*kleb*2^, differ not only in their genetic architecture and core components but in their phylogenetic distribution across *Klebsiella* species. As shown in Fig 1C, these loci are differentially enriched within two major lineages: the KpSC and the *K. oxytoca* species complex (KoSC). Both loci are broadly conserved in KpSC—particularly in *K. pneumoniae* and *K. africana*—which are commonly isolated from the gut, respiratory tract, and bloodstream, and frequently encountered in both clinical and environmental contexts. The high prevalence of T6SSs in these species suggests a reliance on T6SS-mediated antagonism to compete effectively in densely populated and competitive niches, consistent with their pathogenic potential and ecological adaptability. In contrast, KoSC species—including *K. oxytoca*, *K. pasteurii*, *Klebsiella grimontii*, and *Klebsiella michiganensis*—typically retain only T6SS^*kleb*2^ or lack both loci altogether. These bacteria often occupy more stable or specialized environments and tend to function as commensals, facing reduced interbacterial competition or relying on alternative survival strategies. Collectively, the divergent distribution of T6SS^*kleb*1^ and T6SS^*kleb*2^ underscores that T6SS-mediated antagonism is not universally essential across the genus but is shaped by ecological pressures, microbial community complexity, and host associations. In highly competitive environments, the conservation of both loci suggests that they may play complementary and essential roles in interbacterial interactions. Nevertheless, T6SS^*kleb*2^ may also contribute to community stabilization or interactions beyond direct antagonism.

In particular, the function of T6SS^*kleb*2^ has been identified as essential for long-term intestinal colonization, suggesting that this system mediates antagonistic bacterial interactions that can compromise microbiota stability and resilience [54]. Actually, microbial competition mediated by the T6SS of *K. pneumoniae* is more complex, Daniel Storey *et al* identified strain *K. pneumoniae* Kp52145 possess three T6SS loci: locus I (T6SS^*kleb*2^ in our study) contains *vgrG*1 gene, which plays an essential role in *Klebsiella*-mediated killing. In contract, two additional incomplete gene clusters exhibit T6SS-dependent killing activity that varies according to environmental cues [10]. Despite these findings, research on the T6SS in *Klebsiella*, and more specifically on T6SS^*kleb*1^, remains rather limited. T6SS^*kleb*1^ typically encodes key T6SS-related proteins, it often lacks certain core genes, suggesting a role in specialized functions. Our research reveals the presence of numerous orphan T6SS-related genes outside the major loci in *Klebsiella* genomes. This observation further indicates that the functionality of T6SS^*kleb*1^ may be interdependent with T6SS^*kleb*2^ or other loci. Moreover, the presence of multiple *vgrG* and *tssD* paralogs in many genomes suggests redundancy or specialization in effector delivery, as seen by other bacteria like *Vibrio fischeri* [56], or *Pseudomonas aeruginosa* [57]. Future work needs to combine transcriptomics and functional studies, to test whether the *Klebsiella* paralogs are differentially expressed, or functionally interchangeable. Additionally, we identified rare T6SS variants that account for less than 2% of total genomic content. These variants likely represent strain-specific adaptations.

Thus far, only two T6SS effectors, Tle1^KP^ and VgrG4, have been characterized in the *Klebsiella* genus [10,58], emphasizing the significant gap in our understanding of T6SS in this bacterium. This limited knowledge highlights the necessity of comprehensive studies to identify additional T6SS effector and immunity proteins, as well as associated protein families. To address this, we performed a homologous protein search method and positional information to identify 50 total effector proteins and 28 immunity proteins within the genus, which expanded the knowledge of T6SS systems in *Klebsiella*.

We found that there are seven conserved effectors and four immunity proteins common to all *Klebsiella* species. Such conserved proteins may be fundamental to bacterial fitness, perhaps as key players in interbacterial competition or resilience to environmental stressors. Yet, the exact protein mechanisms involved in these processes are still to be elucidated. Besides these conserved proteins, we identified numerous effectors and immunity proteins unique to an individual *Klebsiella* species or strain. For instance, we observed ten effectors that are uniquely found in *K. pneumoniae*, which might be due to its distinct adaptations to environmental pressure. For example, Hcp-ET3 (EFF01522), as described in a previous study, was also isolated from *E. coli* strain PE086 and had antibacterial activity on recipient *E. coli* [27]. This enrichment of unique effectors aligns with *K. pneumoniae*’s broader ecological adaptability, high virulence, and antibiotic resistance. However, the roles of these proteins in *Klebsiella* remain speculative and require further investigation.

Furthermore, we discovered complex relationships among T6SS effector and immunity proteins in our analysis. We found examples in which one immunity protein neutralizes several effector proteins and examples in which one effector protein is targeted by several immunity proteins. This plasticity may be due to adaptation to interbacterial competition polymicrobial environments. This phenomenon is aligned with results reported in *Serratia proteamaculans*, where a T6SS effector, Tre1, targets bacterial essential protein FtsZ through ADP-ribosylation [59], thus blocking cellular division. The immunity protein Tri1 protects against this toxicity via two mechanisms of action, the occlusion of the active site and the enzymatic removal of ADP-ribose modifications. Notably, the former mechanism provides broad protection against non-cognate toxins, highlighting the plasticity and flexibility of effector-immunity networks in interbacterial competition. Similarly, the *Bacteroides* genus has been reported to possess bacterial acquired interbacterial defense (AID) systems that confer the capacity to neutralize a wide repertoire of T6SS effectors from other species. These systems are composed of arrays of orphan immunity genes that have broad-spectrum mechanisms of action that confer resistance to diverse toxins. For example, *Bacteroides fragilis* employs recombinase-associated AID (rAID-1) clusters that enable resistance against its own effectors along with microbial competitors. This underscores the role of interbacterial antagonism in shaping diverse and highly adaptable effector-immunity networks [60].

Experimental approaches to identify T6SS-associated proteins include comparative proteomics [11,15,17,18,61] and transposon mutagenesis [19], whereas computational methods include primarily the search for genes located in proximity to T6SS clusters [20–22,62–64] or genes encoding marker domains [22,23]. While these methods have proven effective, they are very limited in their application, as they do not detect orphan effectors or auxiliary proteins that sit outside of T6SS loci or lack conserved domains. Comparative genomics have been previously used to identify a large family of membrane-disrupting T6SS effectors (Tme), demonstrating the effectiveness of this method [24]. In addition to identifying effectors, our study utilizes comparative genomics to reveal a wide range of T6SS-associated protein families, including regulatory, structural, and auxiliary genes. The broadened perspective allows us to gain important insights on the genomic and functional landscape of T6SS, and its role in bacterial competition and adaptation.

By employing our bioinformatics approach, we successfully uncovered and experimentally validated three T6SS-associated proteins, underscoring the robustness of our method at elucidating novel effectors. We specifically validated proteins harboring DUF3258, DUF3751, and Sel1 domains and found them strongly associated with the presence of T6SS. These proteins displayed cytotoxicity in growth inhibition assays in *E. coli* DH5α, thus confirming their activity as potential T6SS effectors. Phylogenetic analyses of these proteins containing above domains also revealed their predominated distribution in *Klebsiella* and *Escherichia*. These results demonstrate the power of our integrative approach in identifying and characterizing both canonical and orphan T6SS effectors and expanding our knowledge of their ecological and functional roles.

Beyond identifying putative orphan effector proteins, our analysis identified T6SS-associated gene clusters sharing links to bacterial physiology and adaptation. This includes the “Usher-Chaperone” system and its fimbrial components essential for biofilm formation and tropicalization at the host interface. We also identified regulatory and metabolic genes, for example, those involved in sugar transport or gene transcription regulation, that likely impacted T6SS expression and activity. The additional presence of defense-related loci with CRISPR-associated domains signify the implication of T6SS in the coordination of immunity and environmental response mechanisms in bacteria. This study demonstrates that T6SS represents an integrated system with both fundamental influences on bacterial homeostasis and ecological interactions, which forms the basis for investigating new therapeutic approaches directed toward its wider functional network.

In summary, our work presents a comprehensive survey of T6SS loci, effector proteins and their associated gene clusters in the genus *Klebsiella*. T6SS is the key player in bacterial competition, immunity and environmental adaptation and thus our investigation emphasizes the multifaceted and diverse nature of this secretion system. Moreover, the identification of gene clusters related to adhesion, metabolism, and defense systems provides insights into the wider physiological and ecological functions of T6SS. These findings provide a genomic framework for understanding the structural and functional diversity of T6SSs across the *Klebsiella* genus, and offer a foundation for future studies on their ecological roles and potential contributions to bacterial competitiveness or host interactions.

## Materials and methods

### Data acquisition and quality assessment

The comprehensive assemblies from *Klebsiella* genus available in the NCBI Genome database (as of November.1th, 2023) were downloaded. We first performed a rarefaction analysis to evaluate the diversity of T6SS-associated proteins. The rarefaction curve demonstrated that the number of unique T6SS-associated proteins, including effectors, immunity proteins and regulators rapidly approached saturation as the number of genomes increased (S6 Fig). This result indicated that the inclusion of additional genomes would contribute minimal novel T6SS-associated genes. Based on this observation, we implemented a stratified sampling strategy to select a representative subset of genomes for downstream analyses.

For *K. pneumoniae*, sequence typing (ST) was performed using Multilocus Sequence Typing (MLST) via Kleborate v2.3.2 [65] to classify the assemblies, followed by random selection of 10% of genomes from each sequence type for downstream analyses. For other species within the genus, 10% of the genomes were randomly selected. The quality and reliability of the genome were assessed by CheckM v 1.2.2 [66], acquiring a high-quality genome (>99%) and low contamination (<1%).

### Identification of T6SS loci in *Klebsiella* spp.

All assemblies that passed the quality check were subjected genome annotation with Prokka v 1.14.6 [67]. Experimentally validated T6SEs and T6SIs and structural proteins archived in SecReT6 [25] were used as the databased for orthologous searching against the annotated *Klebsiella* proteins with Proteinortho v 6.3.0 [26]. T6SS loci were identified based on the presence of the core components (TssA-M) alongside regional-specific proteins documented in the database. To ensure accurate delineation of T6SS gene loci, a stringent criterion was required: a maximum allowance of five non-T6SS-core genes between two core genes, ensuring their co-localization within the same gene locus on the same contig. Occurrences of identical arrangement patterns exceeding 2% of the total number of genomes were classified as a distinct structural category. Informed by prior study [54] and our own data-derived observations, loci with eight or more core components in T6SS^*kleb*1^ and eleven or more in T6SS^*kleb*2^ are defined as T6SS^*kleb*1^+ and T6SS^*kleb*2^ + respectively, and loci with less than five core components both loci are T6SS-. Also, those with fewer than five in only one are designated as T6SS^*kleb*1^- or T6SS^*kleb*2^-. Those not meeting any of these criteria are considered partial. Genomes carrying both T6SS^*kleb*1^+ and T6SS^*kleb*2^ + loci are designated as T6SS^*kleb*1,2^ + . Structural characteristics of loci were visualized using the ggplot2 v3.5.1 (https://ggplot2.tidyverse.org) package in R studio (version 4.3.3). The proportion of genomes in locus was visualized using the matplotlib [68] package in Python.

### Genus-wide phylogenetic tree construction and T6SS locus typing

A random high-quality representative genome with completeness>99%, contamination<1% and fewer than 50 contigs was chosen for each species to construct the genus-level phylogeny. The phylogenetic tree was built using core gene alignments generated from PhyloPhlAn v 3.1.68 [69] and inferred with IQ-TREE 2 v 2.2.6 [70] based on VT + F + R3 model with the lowest Bayesian Information Criterion (BIC) score. To enhance the robustness of the results, 10,000 bootstrap replicates were conducted. *E. coli* assembly ASM584v2 was used as the outgroup. The phylogenetic tree and gene locus distribution were visualized with iTol [71]. Loci from genomes with a complete T6SS were further typed based on *tssB* genes, which were clustered using CD-HIT v 4.8.1 [72] with 100% similarity and 100% coverage (-c 1 -s 1). The *tssB* sequences were aligned by MAFFT v 7.520 [73], followed by the removal of poorly aligned regions using trimAl v1.4.rev15.build [74] and retained aligned sequences were constructed to phylogenetic tree by FastTree v2.1 [75], and visualized with iTol [71].

### Comparative analysis of T6SS effector and immunity proteins distribution across *Klebsiella* genomes

High-quality genomics data and experimentally validated effector and immunity proteins from the SecRet6 [25] database were homologated to quantify the types and numbers of T6SS effector and immunity proteins in 11 species and *Klebsiella sp*. within the genus to compare their inter- and intraspecific differences. Differences in protein variety and genomic counts were further examined between genomes containing complete T6SS gene clusters (T6SS^*kleb*1,2^+) and those lacking any complete clusters (T6SS-). The data was first normalized by the total number of genomes in each group (T6SS^*kleb*1,2^+ and T6SS-) to account for the genomic counts of homologous effector and immunity proteins. Then, the log2 of the ratio of the normalized counts between two groups was calculated to assess the differences in protein distribution. Statistical significance was subsequently computed based on these normalized values. The heatmap, histogram and statistical analyses were visualized with the pheatmap v1.0.12 (https://CRAN.R-project.org/package=pheatmap) and ggplot2 v3.5.1 (https://ggplot2.tidyverse.org) packages in R studio (version 4.3.3).

### Identification and annotation of VgrG-associated effector-immunity pairs

Previous studies have indicated that VgrG-associated effectors, co-effectors, and immunity proteins were typically encoded downstream of the *vgrG* genes in the adjacent genomic regions. To develop a comprehensive catalog of effectors/immunity proteins, *vgrG* genes were identified using HMMER 3.4 [76] with a *vgrG* sequence as the seed in hmmsearch. Up to three genes downstream *vgrG*s within the same contig in genomes with T6SS^*kleb*1,2^+ and T6SS- were selected and subsequently annotated with Pfam-scan against the full PFam A database [77]. Additionally, a homologous search was performed using Proteinortho v 6.3.0 [26] to compare these genes with experimentally validated effectors and immunity proteins documented in the SecRet6 [25] database. The corresponding protein structural domains of all these genes, paired effector and immunity proteins, were visualized using Cytoscape v 3.10.2 [78].

### Identification and functional annotation of T6SS-associated protein families

Our results indicate that known T6SS-associated proteins, including effectors and immunity proteins are distributed differently between T6SS+ and T6SS- genomes. To investigate protein families potentially linked to T6SS functionality, we focused on those enriched in T6SS+ genomes. The protein sequences from genomes were grouped based on the presence or absence of T6SS at the T6SS^*kleb*1^ and T6SS^*kleb*2^ loci, and clustering was performed separately for two group (i.e., T6SS^*kleb*1^+ and T6SS^*kleb*1^-, T6SS^*kleb*2^+ and T6SS^*kleb*2^-) using CD-HIT v4.8.1 [72] (-c 0.7, -s 0.7). Homologous protein families across different species were analyzed, and the abundance of each protein family in T6SS+ and T6SS- genomes was normalized to the total number of genomes in each group. Statistical analysis of the normalized data was performed using the Chi-square test and protein families significantly associated with different T6SS loci were identified based on specific cutoff: Log2 P/A ≥ 2 & Adjusted *p*-value < 0.05. The homologous grou*p*s were then subjected to annotation with SecReT6 [25] database, Bastion6 [42] and eggNOG-mapper v2 [43], respectively, in order to identify protein families with uncharacterized T6SS-associated functions. The structural domains of these T6SS-associated protein families are further visualized using Cytoscape v 3.10.2 [78] and protein sequences containing DUF3258 (Protein family cluster 28032), DUF3751 (Protein family cluster 4724) and Sel1 (Protein family cluster 8841) domains were further validated *in vitro*. Protein sequences containing DUF3258, DUF3751 and Sel1 in clinical strains were identified, three iterations of Position-Specific Iterated (PSI–BLAST) were performed against the reference protein sequences (a maximum of 500 hits with an expect value threshold of 5 × 10^−3^ were used in each iteration). 500 homologous protein sequences downloaded were aligned by MAFFT v7.520 [73] and gaps were removed from each alignment using trimAl v1.4.rev15.build [74], the maximum-likelihood tree was constructed using IQ-TREE2 v2.2.6 [70] based on the trimmed alignment and visualization in iTol [71].

### Collection and analysis of clinical isolates for T6SS-associated protein validation

To validate our predictions of T6SS-associated protein families, we analyzed clinical isolates of *K. pneumoniae* to identify and confirm the presence of key orthologous genes associated with T6SS functionality. Clinical isolates from patients infected by *K. pneumoniae* were collected in the respiratory department of the First Hospital of Jilin University. Genomic DNA was extracted from isolates using TIANGEN Bacterial Genomic DNA Extraction Kit (Beijing, China). Genomic DNA sample was fragmented by Covaris LE220R-plus (Covaris, USA) to a size of 350 bp for Illumina sequencing and further PCR amplification. PCR products were purified by AMPure XPsystem (Beckman Coulter, Beverly, USA). Subsequently, the library quality was assessed using the Agilent 5400 system (AATI) and quantified by real-time PCR at a concentration of 1.5 nM. Whole-genome sequencing was performed on Illumina platforms at Novogene Bioinformatics Technology Co., Ltd. (Beijing, China) using the PE150 strategy. To ensure data quality, raw sequencing data was processed including de-adapter, removal of low-quality nucleotides and unrecognizable nucleotide (N) and Fastp v 0.23.1 [79] was used to perform basic statistics on the quality of the raw reads. Specific clinical bacterial isolates KP117, KP186, and KP122 were subsequently selected for experimental validation.

### Construction of recombinant plasmids and inducible expression in clinical isolates

The gene of interest, derived from clinical bacterial isolates, was amplified via polymerase chain reaction (PCR). The PCR products were then cleaved with restriction enzymes and ligated into the modified pET28a-tac plasmid, which confers kanamycin resistance. This plasmid is a derivative of the pET28a vector, wherein the T7 promoter has been substituted with the tac promoter to facilitate inducible expression in *E. coli* DH5α cells. The vector retains the His-tag sequence for subsequent protein purification and detection. The digestion-ligation reaction was performed overnight at 16°C using T4 DNA ligase. Subsequently, the ligation mixture was introduced into *E. coli* DH5α competent cells via heat shock transformation. Following kanamycin resistance-based selection, individual transformants were cultured in Lysogeny Broth (LB) medium, and the plasmids were isolated, confirmed by restriction digestion, and sequenced for accuracy.

A single colony of *E. coli* DH5α harboring the recombinant plasmid was selected from a fresh LB agar plate and used to inoculate LB medium supplemented with 30 μg/mL kanamycin. The next day, the cultures were diluted 1:100 into 5 mL of fresh LB medium and incubated with shaking at 220 rpm for 2–3 hours at 37°C until the optical density at 600 nm (OD600) reached 0.6–0.8. One milliliter of each culture was then transferred into three separate 1.5 mL microcentrifuge tubes and centrifuged at 4500 rpm for 2 minutes. The supernatant was discarded, and the bacterial pellets were resuspended in sterile water to adjust the OD600 to 1.0. A series of 5-fold dilutions was prepared, and these dilutions were spotted onto LB agar plates containing 30 μg/mL kanamycin, with and without the addition of 0.2 mM Isopropyl β-D-1-thiogalactopyranoside (IPTG). The plates were incubated at 37°C for 18 hours. Antibacterial activity against *E. coli* DH5α was evaluated through image analysis. The bacterial strains and plasmids employed in this study are described in detail in S5 Table.

### Western blot

Bacterial cultures, both induced and uninduced with IPTG, were harvested and subjected to centrifugation at 12,000 rpm for 2 minutes. The expression of target proteins was analyzed by western blot as described before [80,81]. Briefly, the resulting pellets were resuspended in 50 µl of 1 × SDS-PAGE loading buffer and subsequently boiled in a metal block heater at 100°C for 10 minutes to denature the proteins. The protein samples were then separated by sodium dodecyl sulfate-polyacrylamide gel electrophoresis (SDS-PAGE) using a 10% polyacrylamide gel under a constant voltage of 90V. Following electrophoresis, the proteins were transferred onto a polyvinylidene fluoride (PVDF) membrane at a constant current of 200 mA.

The PVDF membrane was then blocked with 5% non-fat dry milk for 1 hour to prevent non-specific binding. Subsequently, the membrane was incubated with a mouse anti-His monoclonal antibody (proteintech, Cat NO.:66005, 1:3000) overnight at 4°C. After thorough washing to remove unbound antibody, the membrane was probed with horseradish peroxidase (HRP)-conjugated goat anti-mouse secondary antibody (ABclonal, Cat.NO: A5003, 1:3000) for 1 hour at room temperature. Protein detection was carried out using an enhanced chemiluminescence (ECL) kit, and the protein bands were visualized using a gel documentation system (Tanon5200) to evaluate the expression levels.

### Statistical analysis and data representation

To compare the abundance of T6SS-associated proteins, an F-test was first conducted to assess the homogeneity of variances. When the variances were equal, a Student’s t-test was used; otherwise, Welch’s t-test was applied. For comparisons involving more than two groups, the Mann–Whitney U test was performed, and Bonferroni correction was applied to adjust for multiple comparisons. A *p*-value of less than 0.05 was considered as significant. Normality was tested with the Kolmogorov-Smirnov test. All analyses were performed using R studio 4.3.3 and Python.

## Supporting information

S1 Fig Distribution of effector and immunity proteins across *Klebsiella* species. (A) Heatmap displaying the proportions of 28 immunity proteins in the genomes of various *Klebsiella* species. (B) Concentric rings represent the distribution of immunity proteins (imm) across different species. The innermost ring represents 4 immunity proteins found across all species.
(TIF)

S2 Fig Distribution of T6SS immunity proteins in genomes with T6SS^*kleb*1,2^+ and T6SS*-.* (A) Comparison of unique immunity proteins between genomes with T6SS^*kleb*1,2^+ and genomes without any T6SS loci (T6SS-), illustrated by a Venn diagram. (B) Fold-change analysis (log2-transformed) of genomic counts for immunity proteins between T6SS^*kleb*1,2^+ and T6SS- genomes, with a fitted trend line. Immunity proteins are labeled numerically. (C) Histogram of log2-transformed fold changes for all identified immunity proteins, normalized by genome counts (T6SS^*kleb*1,2^ + vs. T6SS-) among the two genome groups. The dotted line represents the mean fold-change value. (D) Comparison of the mean value of immunity proteins per genome between T6SS^*kleb*1,2^+ (n = 2,616) and T6SS- (n = 190). Error bars represent the standard deviation, and statistical significance was assessed using a one-sided Welch’s t-test (****p < 0.0001).
(TIF)

S3 Fig Comparative analysis of effector protein abundance and locus-specific effector repertoires among genomes harboring different T6SS configurations. (A) Comparison of the mean number of effector proteins between genomes carrying both T6SS^*kleb*1^+ and T6SS^*kleb*2^ + loci and those carrying only the T6SS^*kleb*1^ + locus. Error bars represent standard deviations. Statistical significance was assessed using a two-tailed Student’s t-test (****p < 0.0001). (B) Comparison of the mean number of effector proteins between genomes carrying both T6SS^*kleb*1^+ and T6SS^*kleb*2^ + loci and those carrying only the T6SS^*kleb*2^ + locus. Error bars represent standard deviations. Statistical significance was assessed using a two-tailed Welch’s t-test (**p < 0.01). (C) Venn diagram showing the number of protein families significantly associated with the T6SS^*kleb*1^ and T6SS^*kleb*2^ locus, respectively. (D) Venn diagram displaying the total number of predicted and previously characterized effector proteins associated with each locus.
(TIF)

S4 Fig Network analysis of protein families enriched in T6SS^*kleb*1^-associated genomes. The network, constructed using Cytoscape, includes 608 protein families associated with T6SS^*kleb*1^. Node size is proportional to the number of proteins in each family, with colors transitioning from dark to light based on abundance. Edge colors transition from yellow to green to purple based on interaction frequency. Node labels are prioritized by preferred names, followed by eggNOG-mapper annotations and cluster IDs.
(TIF)

S5 Fig Schematic representation of the six gene clusters in T6SS^*kleb*1^ and T6SS^*kleb*2^. Each gene cluster is colored to match the corresponding cluster shown in Fig 4B and 4C. Gray arrows represent additional proteins found in the surrounding genomic regions.
(TIF)

S6 Fig Rarefaction curve showing the relationship between the number of genomes and the unique T6SS-associated genes. Each curve corresponds to a distinct T6SS protein group: effector proteins (purple), immunity proteins (orange), regulators (green) and merged (gray).
(TIF)

S1 Table Summary of data acquisition, species representation, and genomic quality metrics. (XLSX)

S2 Table Detailed annotation information and statistical values for all protein families enriched with T6SS^*kleb*1^, T6SS^*kleb*2^ and T6SS^*kleb*1,2^. (XLSX)

S3 Table Detailed annotation information and statistical values for all protein families enriched with specific T6SS loci configurations. (XLSX)

S4 Table Annotation and statistical values for gene clusters that are associated with T6SS loci. (XLSX)

S5 Table Bacterial strains and plasmids used in this study. (DOCX)
