# Supplementary figures and images for: Genomic profiling and experimental validation of type VI secretion system-associated proteins in Klebsiella
Source: PLoS Genet. 2025 Sep 19;21(9):e1011878. doi: 10.1371/journal.pgen.1011878 (PMC12469244; doi:10.1371/journal.pgen.1011878)

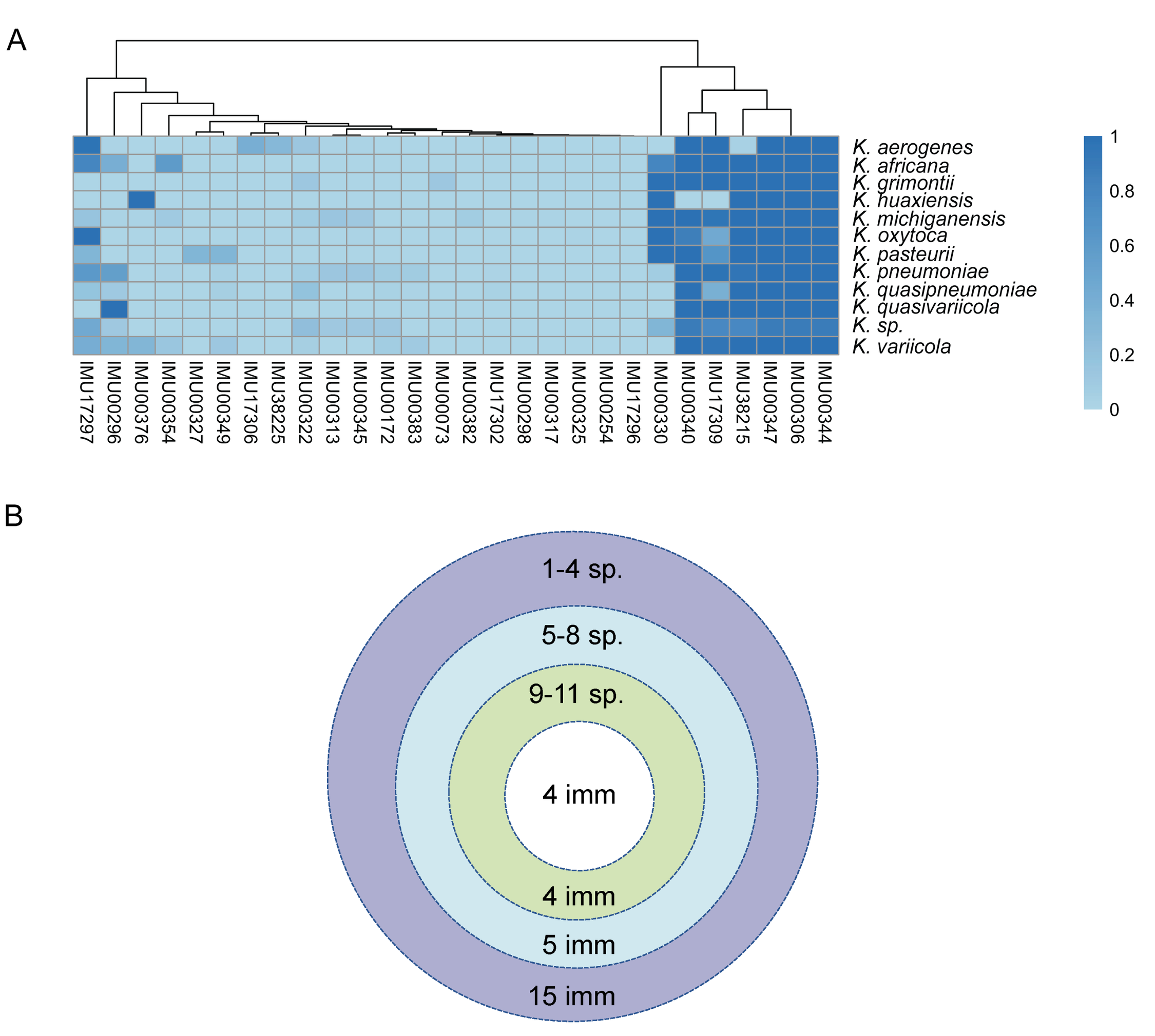

Supplement: S1 Fig — (A) Heatmap displaying the proportions of 28 immunity proteins in the genomes of various Klebsiella species. (B) Concentric rings represent the distribution of immunity proteins (imm) across different species. The innermost ring represents 4 immunity proteins found across all species. (TIF) [file pgen.1011878.s001.tif]

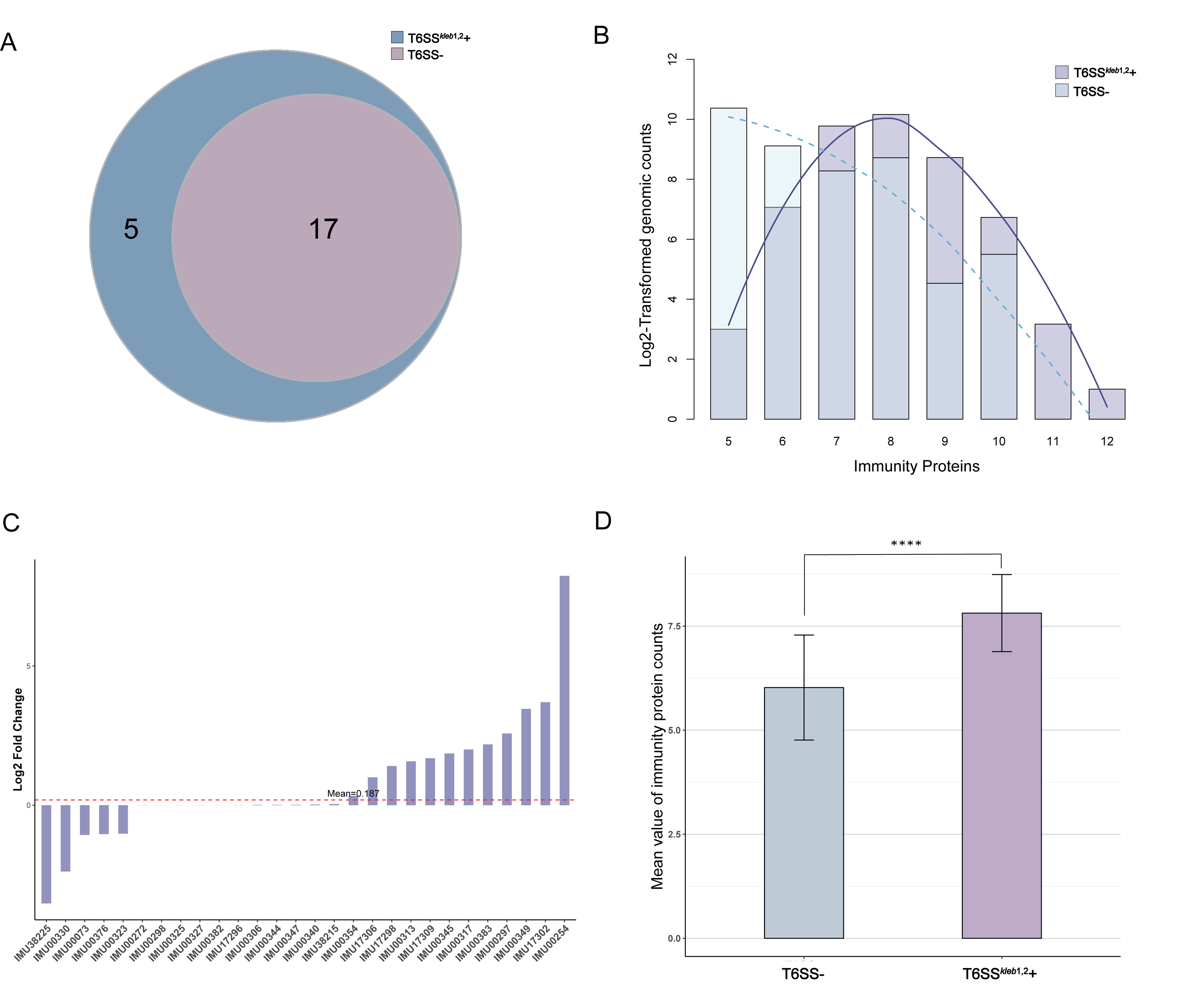

Supplement: S2 Fig — (A) Comparison of unique immunity proteins between genomes with T6SSkleb1,2+ and genomes without any T6SS loci (T6SS-), illustrated by a Venn diagram. (B) Fold-change analysis (log2-transformed) of genomic counts for immunity proteins between T6SSkleb1,2+ and T6SS- genomes, with a fitted trend line. Immunity proteins are labeled numerically. (C) Histogram of log2-transformed fold changes for all identified immunity proteins, normalized by genome counts (T6SSkleb1,2 + vs. T6SS-) among the two genome groups. The dotted line represents the mean fold-change value. (D) Comparison of the mean value of immunity proteins per genome between T6SSkleb1,2+ (n = 2,616) and T6SS- (n = 190). Error bars represent the standard deviation, and statistical significance was assessed using a one-sided Welch’s t-test (****p < 0.0001). (TIF) [file pgen.1011878.s002.tif]

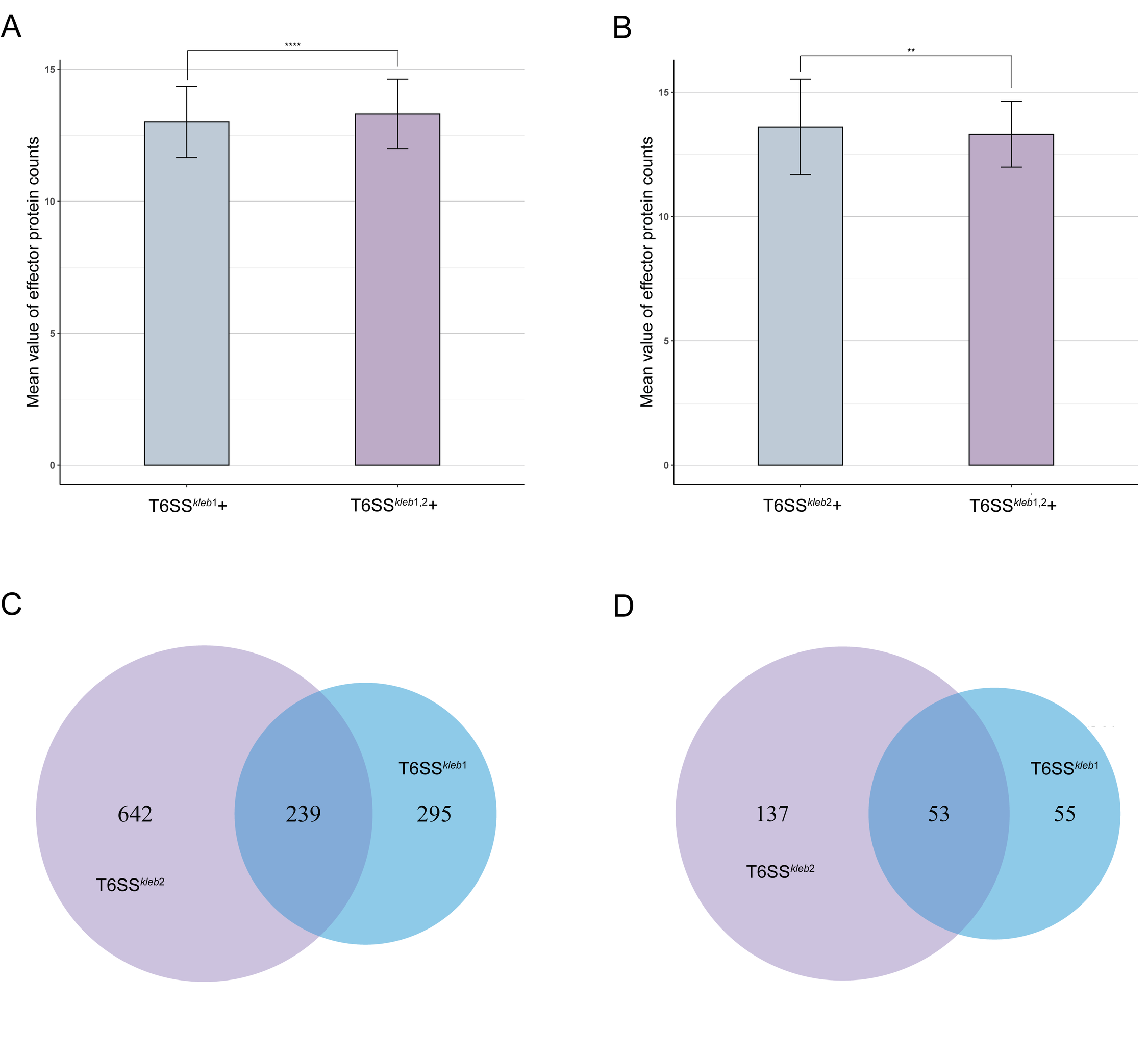

Supplement: S3 Fig — (A) Comparison of the mean number of effector proteins between genomes carrying both T6SSkleb1+ and T6SSkleb2 + loci and those carrying only the T6SSkleb1 + locus. Error bars represent standard deviations. Statistical significance was assessed using a two-tailed Student’s t-test (****p < 0.0001). (B) Comparison of the mean number of effector proteins between genomes carrying both T6SSkleb1+ and T6SSkleb2 + loci and those carrying only the T6SSkleb2 + locus. Error bars represent standard deviations. Statistical significance was assessed using a two-tailed Welch’s t-test (**p < 0.01). (C) Venn diagram showing the number of protein families significantly associated with the T6SSkleb1 and T6SSkleb2 locus, respectively. (D) Venn diagram displaying the total number of predicted and previously characterized effector proteins associated with each locus. (TIF) [file pgen.1011878.s003.tif]

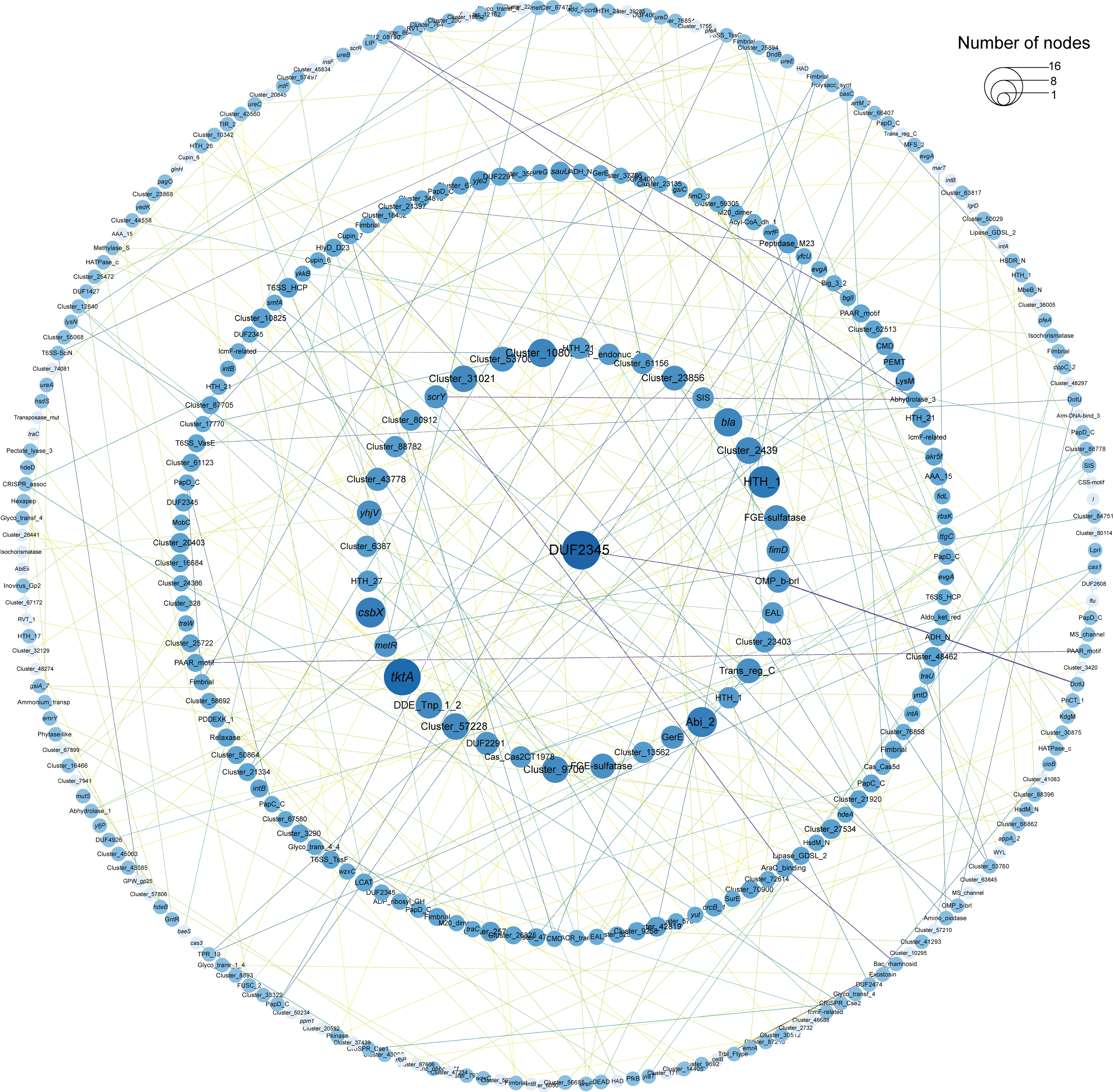

Supplement: S4 Fig — The network, constructed using Cytoscape, includes 608 protein families associated with T6SSkleb1. Node size is proportional to the number of proteins in each family, with colors transitioning from dark to light based on abundance. Edge colors transition from yellow to green to purple based on interaction frequency. Node labels are prioritized by preferred names, followed by eggNOG-mapper annotations and cluster IDs. (TIF) [file pgen.1011878.s004.tif]

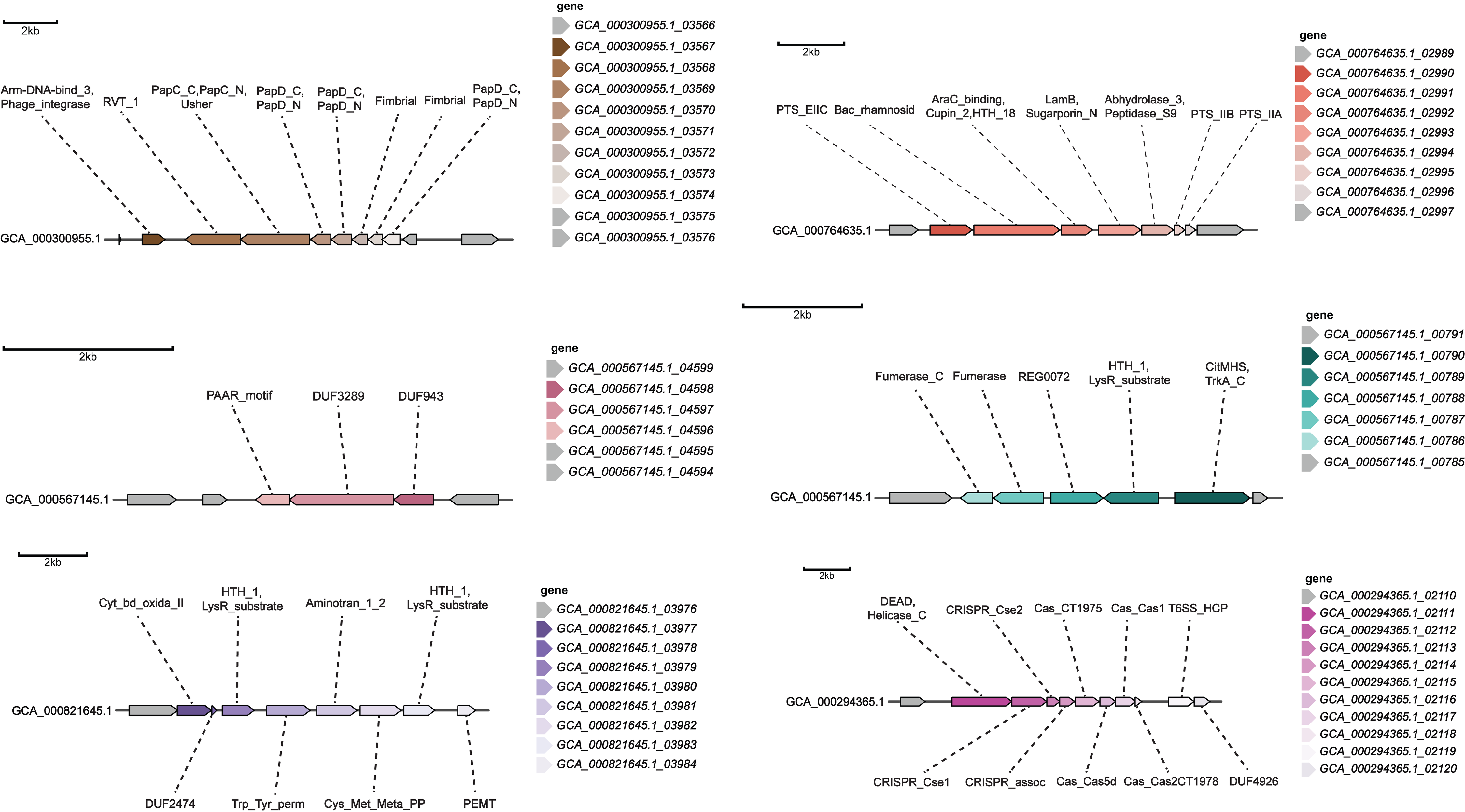

Supplement: S5 Fig — Each gene cluster is colored to match the corresponding cluster shown in Fig 4B and 4C. Gray arrows represent additional proteins found in the surrounding genomic regions. (TIF) [file pgen.1011878.s005.tif]

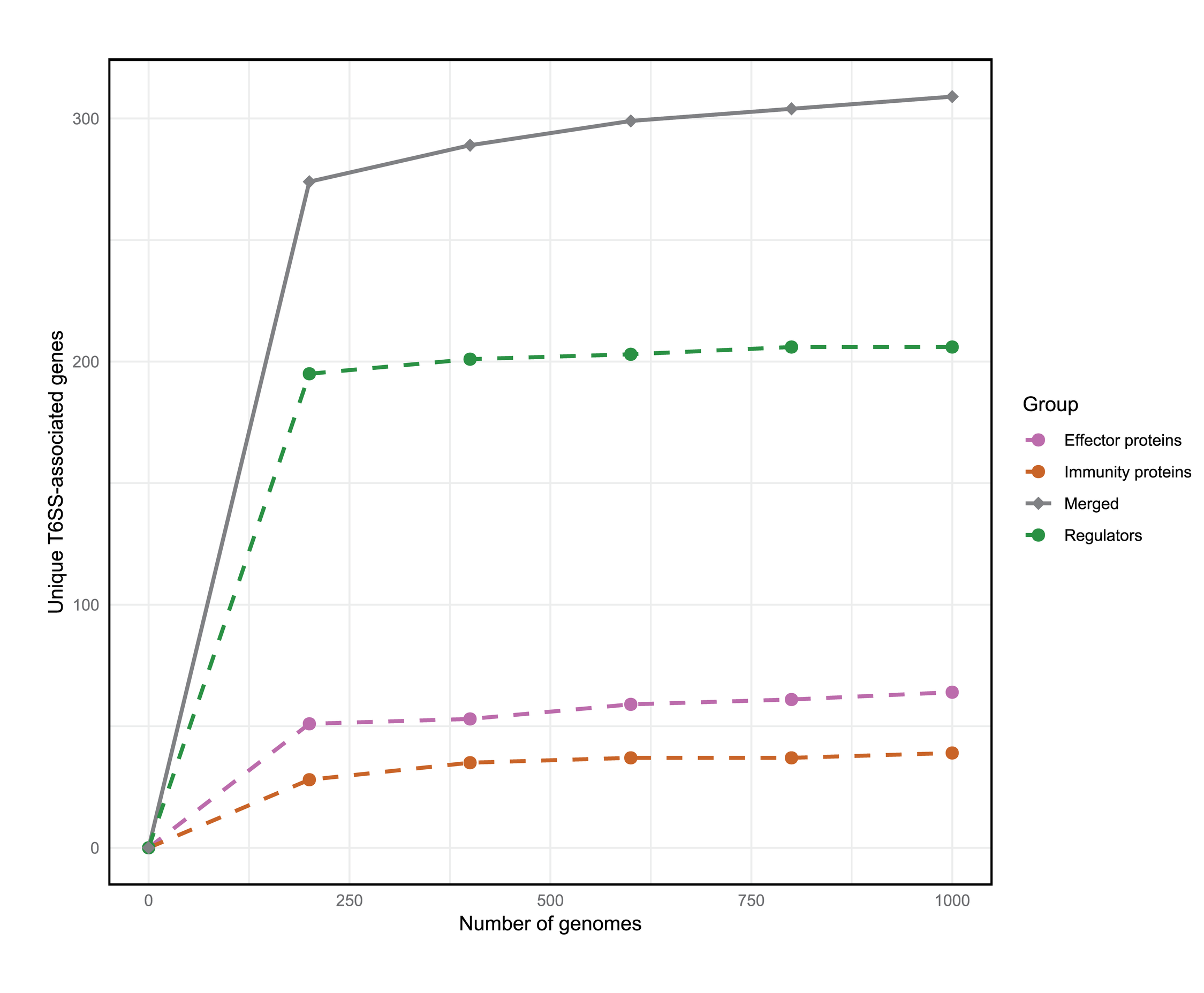

Supplement: S6 Fig — Each curve corresponds to a distinct T6SS protein group: effector proteins (purple), immunity proteins (orange), regulators (green) and merged (gray). (TIF) [file pgen.1011878.s006.tif]
