# Supplementary material for: Genomic profiling and experimental validation of type VI secretion system-associated proteins in Klebsiella
Source: PLoS Genet. 2025 Sep 19;21(9):e1011878. doi: 10.1371/journal.pgen.1011878 (PMC12469244; doi:10.1371/journal.pgen.1011878)
Supplement: S5 Table — (DOCX) [file pgen.1011878.s011.docx]

S5.1 List of strains and plasmids

| Strains or Plasmids | Source | Identifier |
| --- | --- | --- |
| *E. coli* |  |  |
| DH5α | [1] | Cat# KTSM101L |
| Strains |  |  |
| KPN117 | Clinical isolate | N/A |
| KPN122 | Clinical isolate | N/A |
| KPN186 | Clinical isolate | N/A |
| Plasmids |  |  |
| pTAC | KmR, a pET28a-based plasmid harboring P_TAC_ | N/A |
| pTAC DUF3258 | This study | N/A |
| pTAC DUF3751 | This study | N/A |
| pTAC Sel1 | This study | N/A |

S5.2 PCR primer list

| Primers | Sequence (Restriction enzyme sites are underlined) | Note |
| --- | --- | --- |
| DUF3258 -F | 5’-CGCGGATCCATGTCACAGGCTTATGAAGCA-3’ | DUF3258 5’BamHI |
| DUF3258 -R | 5’-ACGCGTCGACTCAATAAATATACCTGCGCCCG-3’ | DUF3258 3’ SalI |
| DUF3751 -F | 5’-cgcGGATCCATGAGCACAAAATTTTATACCCTGC-3' | DUF3751 5’BamHI |
| DUF3751 -R | 5’-ACGCGTCGACTTAATAGCCAATTGCAAGATATGAGC-3' | DUF3751 3’ SalI |
| Sel1 -F | 5’-CCGGAATTCATGAAAATGCGTTATGTCCTGG-3’ | Sel1 5’ BamHI |
| Sel1 -R | 5’-ACGCGTCGACCTATTCCTCTGTCTTTACCAATTCTTG-3’ | Sel1 3’ SalI |

1. Hanahan D. Studies on transformation of Escherichia coli with plasmids. Journal of molecular biology. 1983; 166:557-80. <https://doi.org/10.1016/s0022-2836(83)80284-8> PMID: 6345791
